# Use of tranexamic acid in dogs with primary immune thrombocytopenia: A feasibility study

**DOI:** 10.3389/fvets.2023.946127

**Published:** 2023-03-22

**Authors:** Gerard Olivares, Mellora Sharman, Rachel Miller, Caroline Kisielewicz, Mayank Seth

**Affiliations:** ^1^Department of Small Animal Internal Medicine, Animal Health Trust, Newmarket, Suffolk, United Kingdom; ^2^Department of Small Animal Internal Medicine, Eastcott Veterinary Referrals, Part or Linnaeus Veterinary Limited, Swindon, United Kingdom; ^3^Department of Small Animal Internal Medicine, Dick White Referrals, Cambridgeshire, United Kingdom; ^4^Department of Small Animal Internal Medicine, Pride Veterinary Centre, Derby, United Kingdom

**Keywords:** tranexamic acid, canine, bleeding score, immune thrombocytopenia (ITP), antifibrinolytics

## Abstract

**Objective:**

The aim of this feasibility study is to evaluate the use of tranexamic acid and its safe use alongside standard therapy in dogs with primary immune thrombocytopenia (ITP).

**Design:**

This is a cohort feasibility study involving 10 dogs diagnosed with primary ITP that received standard therapy for ITP including corticosteroids, a single dose of vincristine, and omeprazole. Dogs were randomly divided into either the control group (*n* = 6) or the group receiving tranexamic acid (TXA group, *n* = 4).

**Key findings:**

The mean time from the start of treatment until remission was 5 days in the TXA group and 6 days in the control group (*P* = 0.69). Two dogs, one in each group, did not achieve remission. Clinical bleeding scores were not significantly different between both groups (*p* = 0.43), and the median blood volume administered was 37.5 ml/kg for the TXA group and 9.72 ml/kg for the control group (*p* = 0.084). Three out of the four dogs receiving TXA of 20 mg/kg IV started vomiting within 15 min of administration and were given a reduced dose of 15 or 10 mg/kg IV.

**Conclusion:**

Tranexamic acid did not confer a clinical benefit in this small cohort study and was associated with a high incidence of vomiting. This study provides useful information for the design of future trials in dogs with ITP receiving tranexamic acid including outcome measures and safety.

## Introduction

Immune thrombocytopenia (ITP) is a common cause of severe thrombocytopenia in dogs (1), and glucocorticoids are the mainstay treatment for primary ITP (2). Vincristine and human intravenous immunoglobulin have been documented to shorten the duration of severe thrombocytopenia in dogs with primary ITP compared to glucocorticoids alone (3, 4). In dogs with ITP, reported short-term survival rates ranged from 74 to 84% 0.5 (5, 6). Until therapy is effective, spontaneous hemorrhage may develop, particularly in dogs with platelet concentrations of <30 × 10^9^/L (5). The presence of melena and increased blood urea nitrogen concentration, both reflecting gastrointestinal blood loss, are associated with an increased risk of mortality for dogs with ITP (6).

There is robust evidence in human literature that antifibrinolytic agents, which prevent plasminogen-mediated degradation of fibrin, are effective at reducing blood loss and the need for blood transfusions during surgical procedures, nose bleeds, or menstruation (7). Antifibrinolytic agents have been used in people with ITP, although the available information is limited to case reports or small non-controlled studies (8).

A recent clinical trial in people undergoing treatment for hematologic malignancy (A-TREAT Trial) (9) indicated that the prophylactic use of tranexamic acid has no protective effect against bleeding. In clinical veterinary medicine, antifibrinolytic agents have shown to be effective in reducing post-operative bleeding in greyhounds (10), while the safety and efficacy of TXA have been assessed in additional recent studies (11, 12). However, to the authors' knowledge, there are no studies in dogs with thrombocytopenia receiving anti-fibrinolytics. As occurs in people, a common occurrence in humans, any treatment that could reduce reliance on blood transfusion would have major implications such as reducing the duration and cost of hospitalization. This is especially important in dogs since platelet products have limited availability and can be cost-prohibitive.

The main aim of this feasibility study was to generate pilot data for future studies as well as for the safety of the administration of tranexamic acid in a group of dogs with ITP. We hypothesized that, for dogs with ITP, antifibrinolytics given in addition to conventional therapy would reduce the risk of spontaneous bleeding, thereby reducing the need for blood transfusions. A secondary hypothesis was that this would result in more rapid improvement in platelet numbers due to a reduction in ongoing platelet consumption, thus reducing hospitalization times.

## Methods

### Study population

Client-owned dogs diagnosed with primary ITP who presented to three participating referral hospitals between 2017 and 2019 were enrolled in the study. The study protocol was approved by the clinical research and ethics committee at the primary organizing clinic (26–2017). An Animal Test Certificate was obtained from the UK Veterinary Medicines Directorate for the use of tranexamic acid (Cyklokapron^®^ Injectable).

### Inclusion criteria

Dogs with a presumptive diagnosis of primary ITP were included in the study if their owners gave informed consent and if the platelet count was <40 × 10^9^/L upon enrollment. A diagnosis of primary ITP was made after the exclusion of secondary ITP and other causes of thrombocytopenia. Diagnostic investigations included physical examination, CBC, serum biochemistry, thoracic and abdominal imaging, and serologic testing for *Angiostrongylus vasorum, Anaplasma spp*, and *Ehrlichia spp* plus any other infectious agents based on the discretion of the clinician and individual travel history of the patient. Dogs were excluded from the study if they had received corticosteroids or any other immunosuppressive agent for more than 48 h before study enrollment.

### Study design

This is a cohort feasibility study. The assignment into treatment groups was randomized. The primary measures of the study were the time required to achieve a platelet count of >40 × 10^9^/L, differences in clinical bleeding scores, the duration of hospitalization, and the amount of blood transfused and long-term survival (3 months).

### Concomitant treatments

All dogs were treated with prednisolone (5 mg tablets, Dechra Ltd. 2–3 mg/kg) PO q 24 h or dexamethasone (Dexadreson^®^ injectable 2 mg/ml, MSD Animal Health UK Ltd. 0.15–0.3 mg/kg) IV q 24 h. A single dose of vincristine (Oncovin ^®^ injectable 1 mg/ml, Vincasar PFS. 0.02 mg/kg) IV was also administered. All dogs received omeprazole (Omeprazole sodium injectable 40 mg/ml. 1 mg/kg) IV q 12 h. These treatments were initiated as soon as the clinical diagnosis of ITP was made and within 24 h of the initial presentation. Independent of the treatment group, additional therapies including intravenous fluid therapy, blood products, analgesics, or prophylactic antibiotics were administered at the discretion of the attending clinician. Treatment with other immunosuppressive agents was not permitted during the first 7 days of the study.

### Treatment group

Patients in the treatment group received tranexamic acid (20 mg/kg) IV q 8 h administered as slow infusion (over 15–20 min). Treatment with tranexamic acid was initiated as the clinical diagnosis of ITP was made and within 24 h of the initial presentation. If emesis occurred within 15 min of drug administration, the dose of tranexamic acid was reduced (15 mg/kg) IV q 8 h for subsequent administrations. If emesis occurred at this dose, the dose of tranexamic acid was further reduced (10 mg/kg) IV q 8 h for the subsequent administrations. If emesis occurred at this dose, treatment with tranexamic acid was discontinued.

### Response monitoring

All dogs were monitored daily using a validated daily canine bleeding assessment tool in dogs with ITP (DOGiBAT) (13). Briefly, the bleeding severity of nine different anatomic sites (skin, catheter/venipuncture site, oral mucosa, ocular, nasal, gastrointestinal, urinary, pulmonary, and intracranial) was assessed. The evaluation was conducted by the attending clinician (small animal internal medicine specialists or residents). A numeric score was assigned based on the bleeding severity grade, and a total score out of 18 was given.

Daily measurement of PCV/total solids and platelet count was conducted. Platelet counts were performed either by automated or manual count by the attending clinician. Remission was defined as having a platelet count of ≥ 40 × 10^9^/L. An automated platelet count was required for confirmation. Any other investigations were conducted at the discretion of the attending clinician. All dogs were monitored for potential adverse effects of the drugs administered, and any adverse events were recorded.

### Statistical analyses

The cross-sectional data were compared by means of the Mann–Whitney *U*-test and Fisher's exact test. The repeated measures of data (daily bleeding scores and platelet count) were analyzed by using the summary method approach (14). The time-to-event data (time to reach target platelet count, time to hospital discharge, and patient survival over 3 months) were analyzed by using the log-rank and the Kaplan–Meier curves. A *p*-value of < 0.05 was considered statistically significant.

## Results

A total of 10 dogs were included, of which six were assigned to the control group and four to the tranexamic acid group (TXA). Six dogs were female neutered, two were male neutered, one was a female entire, and one was a male entire. There were two mixed-breed dogs and eight purebreds (three English springer spaniel, two English cocker spaniel, one rottweiler, one toy poodle, and one labrador). There were no significant differences in age, weight, or relevant initial clinical and laboratory findings (Table 1). A clinical assessment at the baseline showed petechiae and/or ecchymosis (*n* = 9), oral bleeding (*n* = 8), ocular bleeding (retinal or hyphema) (*n* = 6), hematuria (macroscopic and microscopic) (*n* = 6), bleeding from venipuncture site (*n* = 4), suspected pulmonary bleeding (*n* = 1), and suspected intracranial bleeding (*n* = 1).

**Table 1:** Baseline characteristics in the 10 included dogs with immune thrombocytopenia in the tranexamic acid group (TXA) and the control groups.

**Characteristics**	**TXA (*n* = 4)**	**Control (*n* = 6)**	***p*-value**
Age-years	8.5 (3–12)	6 (3–11)	0.38
Weight (kg)	12 (8.5–52.6)	16.1 (5.9–33.8)	0.67
Clinical bleeding score (out of 18)	7.0 (6–11)	7.5 (5–10)	0.83
PLT count (PLT/μl)	2.0 (1–4)	7.5 (0–20)	0.163
HCT (L/L)	0.23 (0.12–0.27)	0.27 (0.2–0.57)	0.28
Total solids (g/L)	56.5 (46–68)	54.5 (40–66)	0.59
BUN (mmol/L)	4.3 (2.9–16)	8.6 (6.1–11.2)	0.21
Melena	4/4	4/6	0.467
Gender (male–female)	2–2	1–5	

The results are presented as median and range (in brackets). Platelets (PLT).

### Response to treatment

**Figure 1:**
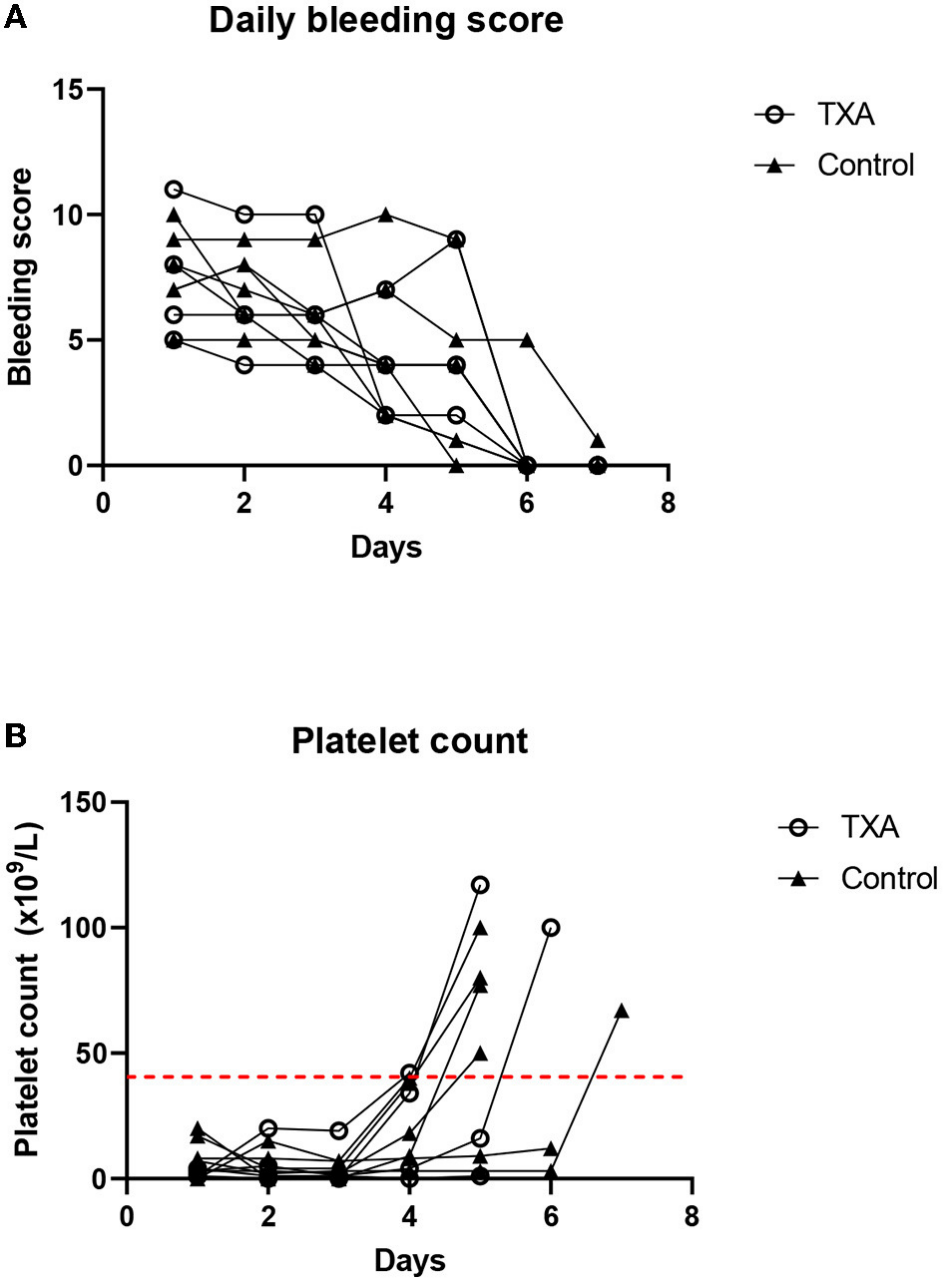
**(A)** The daily bleeding score for individual dogs in the tranexamic acid (TXA) and the control group. **(B)** Daily platelet count for individual dogs in the TXA and the control groups. The dashed red line indicates the cutoff for remission (>40 × 10^9^/L).

Subcutaneous bleeding and gastrointestinal tract hemorrhage were the major sources of blood loss in dogs that required blood products. The daily clinical bleeding score was not significantly different between groups (*P* = 0.43). The bleeding score of all survivors was found to decrease compared to that of the baseline and was ≤4 at the time of remission (Figure 1A). For the two cases that died during the study period, the clinical bleeding score was equal to or higher than that of the baseline. The daily platelet score was not significantly different between groups (*P* = 0.33) (Figure 1B).

A total of six dogs received a transfusion of packed red blood cells (PRBCs), and one dog received both fresh whole blood and PRBCs. The median volume of blood products administered was 37.5 ml/kg for the TXA group and 9.72 ml/kg for the control group. The transfusion requirements were not significantly different between both groups (*P* = 0.084).

### Outcome

**Figure 2:**
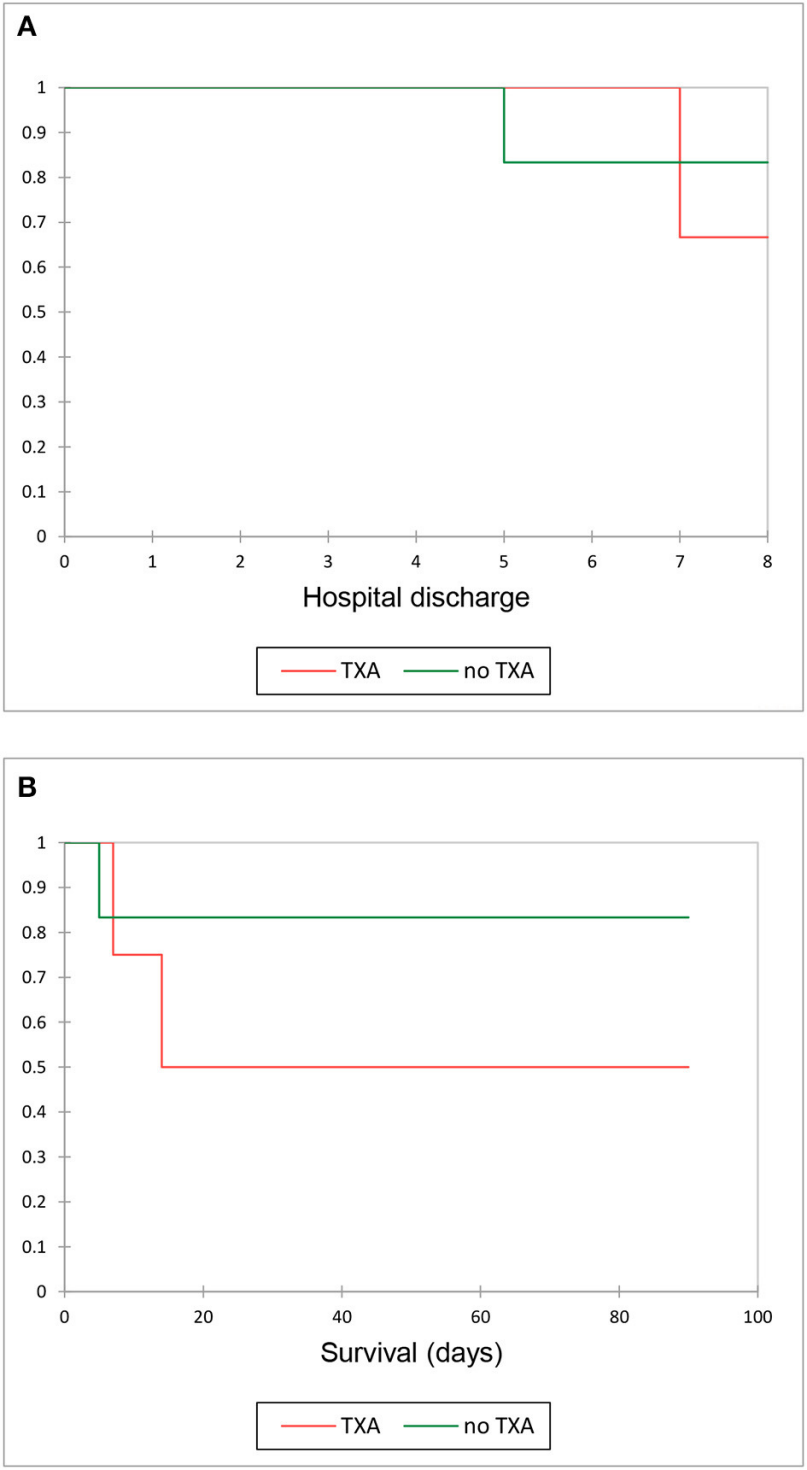
**(A)** Kaplan–Meier curve illustrating survival to hospital discharge in the tranexamic acid group (TXA) and control groups. **(B)** Kaplan– Meier curve illustrating survival 3 months after discharge in the TXA and control groups.

In total, two dogs, one in each group, did not achieve remission and were euthanized due to an ongoing requirement for blood transfusions. The overall mean lag time from the start of treatment until platelet count increased to ≥ 40 × 10^9^/L was 5.0 days in the TXA group and 6.0 days in the control group (*P* = 0.69) (Table 2). The mean time to hospital discharge was 7.0 days in the TXA group and 5.0 days in the control group (*P* = 0.81) (Figure 2A). One dog in the tranexamic acid group was euthanized 14 days after she was discharged from the hospital by the referring veterinarian with a suspected ITP relapse. The rest of the dogs (7/10) were alive at 3 months after diagnosis, and this was not different between the groups (*P* = 0.35) (Figure 2B).

**Table 2:** Outcomes for dogs with immune thrombocytopenia in the tranexamic acid (TXA) and the control groups.

**Characteristics**	**TXA (*n* = 4)**	**Control (*n* = 6)**	***p*-value**
Remission	3/4	5/6	1.0
Days to remission	5.0	6.0	0.69
Mean clinical bleeding score (out of 18)	6.75 (4–10)	5.83 (4–9)	0.43
Units of blood products	3.25 (1–8)	1 (0–3)	0.15
Volume of blood products (ml/kg)	37.5 (17–75)	9.72 (0–36)	0.084

Data are presented as mean for days to remission and clinical bleeding score, median for units and volume of blood products, and range (in brackets).

### Adverse effects

A total of three out of four dogs in our study experienced vomiting during the IV infusion of tranexamic acid at a dose of 20 mg/kg. Following dose reductions, two out of these three dogs still vomited at a dose of 15 mg/kg IV, with one of the dogs vomiting even at the dose of 10 mg/kg necessitating discontinuation altogether. There were no identifiable immediate or delayed adverse drug reactions to the other administered drugs.

## Discussion

In our study, the mean platelet recovery time was 5 and 6 days for the TXA group and the control group (*p* = 0.69), respectively. This is similar to the recovery time reported in studies evaluating the use of standard therapy with prednisolone and vincristine (3). Other variables such as the clinical bleeding score, the volume of blood products, and the status at 3 months were not found to be significantly different between both groups.

The volume of blood products received by the TXA group was larger than that of the control group (37.5 ml/kg for the TXA group and 9.72 ml/kg for the control group), but was not significantly different. The cause for this remains uncertain but, given the low number of cases included and the lack of statistical power, this may represent a type II error, and further studies are required to elucidate this.

Of all the outcomes studied here, we believe that the most relevant ones are the daily changes in clinical bleeding score and the volume of blood products used. While the utility of tranexamic acid in dogs with ITP cannot be concluded on this analysis, a large clinically relevant benefit seems unlikely. A consideration for future studies would be to select a population of dogs with ITP with more severe disease. This could include dogs with a high clinical bleeding score or with active bleeding. Certainly, the latter was the definition used in a study of 12 people with ITP (8), in which the administration of tranexamic acid was associated with a cessation of bleeding.

Overall, the survival rate of our study is in line with a previous cohort study in which 84% of dogs with ITP survived to discharge (6). In total, two dogs, one in each group, did not complete the study and were euthanized due to the ongoing requirement of blood transfusions. In these two euthanized dogs, the bleeding score severity remained increased in comparision to those who survived during hospitalization, compatible with higher disease severity.

Although vomiting was self-limiting, the incidence of vomiting especially in a compromised patient may lead to complications. The use of a 20 mg/kg IV dose in this study was based on previous studies where this dose was well tolerated and found to be both safe and effective (11). A recent pharmacokinetic study of TXA in dogs showed that 10 mg/kg IV and 20 mg/kg IV both improved maximum clot strength and reduction of clot lysis relative to the baseline (12). However, only treatment with 20 mg/kg IV resulted in plasma TXA levels above a threshold that allowed a complete inhibition of fibrinolysis in dogs, and the antifibrinolytic effects also persisted for a longer duration than at the lower dose (12). Given the relatively high incidence of vomiting in our study at 20 mg/kg, future studies might focus on exploring effective and safer administration options for the TXA group, such as more frequent administration of tranexamic acid of 10 mg/kg IV or administration of an IV loading dose, followed by oral drug administration. Another consideration is whether lower doses of antifibrinolytic agents would be sufficient to inhibit fibrinolysis to a clinically relevant degree in a patient with thrombocytopenia rather than the hyperfibrinolytic models which have been used for dosing studies to date.

Viscoelastic testing was not performed in this study in part due to limited availability at study sites. While it would be interesting to assess the global coagulation properties and response to antifibrinolytic therapy in dogs with ITP, protocols to do this have not been standardized and the purpose of this small study was to focus on the overall clinical effect.

This study is one of the first to utilize a recently published clinical bleeding score (13). The system was deemed to be easily applicable by staff across several busy clinical sites supporting its ongoing use in future studies.

In conclusion, this cohort feasibility study provides useful information for the design of future trials about tranexamic acid given to dogs with ITP including outcome measures and safety. Tranexamic acid did not confer a significant clinical benefit in this small cohort and was associated with a high incidence of vomiting. Future studies with larger, more targeted, patient populations and refined dosing protocols are needed to draw more robust conclusions about the use of antifibrinolytics in dogs with ITP.

## Data availability statement

The raw data supporting the conclusions of this article will be made available by the authors, without undue reservation.

## Ethics statement

The animal study was reviewed and approved by Animal Health Trust Ethics Committee. Written informed consent was obtained from the owners for the participation of their animals in this study.

## Author contributions

All authors contributed to the conception and design of the study. All authors contributed to data collection, manuscript revision, read, and approved the submitted version.
